# Association between *DAPK1* Promoter Methylation and Cervical Cancer: A Meta-Analysis

**DOI:** 10.1371/journal.pone.0107272

**Published:** 2014-09-30

**Authors:** Jiaqiang Xiong, Ya Li, Kecheng Huang, Meixia Lu, Hao Shi, Lanfang Ma, Aiyue Luo, Shuhong Yang, Zhiyong Lu, Jinjin Zhang, Lilan Yang, Shixuan Wang

**Affiliations:** 1 Department of Obstetrics and Gynecology, Tongji Hospital, Tongji Medical College, Huazhong University of Science and Technology, Wuhan, Hubei, China; 2 Department of Epidemiology and Biostatistics, and the Ministry of Education Key Lab of Environment and Health, School of Public Health, Tongji Medical College, Huazhong University of Science and Technology, Wuhan, Hubei, China; 3 Department of Obstetrics and Gynecology, The First Affiliated Hospital of Nanchang University, Nanchang, Jiangxi, China; Georgetown University, United States of America

## Abstract

**Background:**

Death-associated protein kinase1 (*DAPK1*) is an important tumor suppressor gene. DNA methylation can inactivate genes, which has often been observed in the carcinogenesis of cervical cancer. During the past several decades, many studies have explored the association between *DAPK1* promoter methylation and cervical cancer. However, many studies were limited by the small samples size and the findings were inconsistent among them. Thus, we conducted a meta-analysis to assess the association between *DAPK1* promoter methylation and cervical cancer.

**Methods:**

We systematically searched eligible studies in the PubMed, Web of Science, EMBASE and CNKI databases. Using meta-regression, subgroup analysis and sensitivity analysis, we explored the potential sources of heterogeneity. The odds ratio (OR) and 95% confidence interval (95% CI) were calculated by Meta-Analysis in R.

**Results:**

A total of 15 studies from 2001 to 2012, comprising 818 tumor tissues samples and 671 normal tissues samples, were analyzed in this meta-analysis. The frequencies of *DAPK1* promoter methylation ranged from 30.0% to 78.6% (median, 59.3%) in cervical cancer tissue and 0.0% to 46.7% (median, 7.8%) in normal cervical tissue. The pooled OR was 19.66 (95%CI = 8.72–44.31) with the random effects model, and heterogeneity was found through the sensitivity analysis. The *I^2^* = 60% (*P* = 0.002) decreased to *I^2^* = 29.2% (*P* = 0.144) when one heterogeneous study was excluded, and the pooled OR increased to 21.80 (95%CI = 13.44–35.36) with the fixed effects model.

**Conclusion:**

The results suggested a strong association between *DAPK1* promoter methylation and cervical cancer. This study also indicated that *DAPK1* promoter methylation may be a biomarker during cervical carcinogenesis that might serve as an early indication of cervical cancer.

## Introduction

Cervical cancer is the third most common cancer, after breast and colorectal cancer, among women worldwide, with 529,500 estimated new cases and 275,000 deaths in 2008 according to Ferlay et al. [1]. The development of invasive cervical cancer is a gradual process that occurs over a long period, from cervical intraepithelial neoplastic (CIN) lesions to cervical cancer. Thus, it is critically important to detect precancerous lesions to prevent the development of cervical cancer. Although infection with the human papillomavirus (HPV) is an accepted major risk factor for cervical cancer [2], only a small proportion of HPV infected patients develop invasive cervical cancer [3]. Other risk factors may also contribute to the genesis of this cancer type.

Hypermethylation of the promoter regions of tumor suppressor genes can cause gene inactivation, which is important in the pathogenesis of cancers, and usually occurs in the early stages of cancer development in various types of cancer, including cervical cancer [4], [5]. DNA rmethylation is an early event in carcinogenesis, and is often related to a transcriptional block and the loss of a relevant protein [6]. Because *DAPK1* is an important tumor suppressor gene that has been studied extensively, we performed a meta-analysis to assess the association between *DAPK1* promoter methylation and cervical cancer.

## Materials and Methods

### Study search and selection criteria

We systematically reviewed the studies of *DAPK1* promoter methylation in cervical cancer, and attempted to find the eligible studies within PubMed, EMBASE, Web of Science and CNKI, using various combinations of Medical Subject Headings (MeSH) and non-MeSH terms. The keywords were “cervical cancer”, “*DAPK1*”, and “methylation”, while the search strategy was performed in PubMed with “uterine cervical neoplasms” (MeSH), “*DAPK1*”and“methylation”. The study was conducted till November 1, 2013 without any language limitation.

The studies for inclusion in this meta-analysis had to meet the following standards: (i) the studies assessed the association of *DAPK1* methylation and cervical cancer, (ii) the studies provided detailed information about the frequency of *DAPK1* methylation for both the cancer group and the normal control group, (iii) methods for the detection of *DAPK1* methylation were limited to the methylation-specific polymerase chain reaction (MSP) and real-time quantitative polymerase chain reaction (QMSP).

Studies were excluded based on the following criteria: (i) the studies did not have a normal group (control group), (ii) the raw data could not be isolated from the studies in which the cancer group (case group) also contained individuals with various types of precancerous lesions such as Atypical Squamous Cells of Undetermined Significance (ASCUS), Low-grade Squamous Intraepithelial Lesions (LSIL), and High-grade Squamous Intraepithelial Lesions(HSIL), (iii) a case-control study did not feature the frequency of *DAPK1* methylation.

## Data Extraction

Two authors independently conducted the extraction of data from the selected studies. The extracted information contained the following: first author's name, publication year, the patients' ethnicities, the methods used in the measurement of *DAPK1* methylation, the tissue source of the control group, the mean age of the case group, and the number of participants in the case and control groups. All the information was verified by three reviewers.

To assess the quality of the studies, The Newcastle–Ottawa scale (NOS) (http://www.ohri.ca/programs/clinical_epidemiology/oxford.asp) was implemented for quality assessment of observational studies. The NOS is a quality assessment tool which is often used for nonrandomized studies, specifically case-control and cohort studies, included in systematic reviews. It has also been widely used in systematic reviews of nonrandomized studies by The Cochrane Collaboration.

There is a maximum of nine ‘stars’ for each item: four stars to the selection of the study groups, two stars to the comparability of the groups, and three stars to the ascertainment of the outcome of interest. The evaluation was performed independently by two reviewers. Studies with quality scores greater than or equal to 6 were included.

### Statistical analysis

The ORs and 95% CIs were calculated to assess the association between *DAPK1* promoter methylation and cervical cancer risk. The *x* ^2^-based Cochran Q statistic test and *I* ^2^ statistics were used to test the heterogeneity among the included studies [7]. Significant heterogeneity was confirmed if *P*<0.05; *I* ^2^>50% was also considered to demonstrate significant heterogeneity [8]. Then, a random effects model (the DerSimonian-Laird estimator) was used to calculate the pooled ORs; otherwise, a fixed-effects model (the Mantel-Haenszel method) was applied [9]. A meta-regression (restricted maximum-likelihood estimator method) was employed to explore the source of the heterogeneity. Furthermore, a subgroup analysis was performed to evaluate the source of the heterogeneity, and t^2^ was used to determine how much heterogeneity could be explained by subgroup differences. A sensitivity analysis was used to find relatively poor-quality studies by the omission of a single study at a time and to see whether a particular omission could affect the overall OR value. The funnel plots [10] and Egger's test were used to evaluate publication bias. The fail-safe number was also an indicator to assess publication bias. An asymmetric plot suggested a possible publication bias and the *P* value of Egger's test less than 0.05 was considered to be representative of a statistically significant publication bias [10].

All statistical analyses were calculated with the Meta package (version 2.5–1) in R (version 3.0.1; http://www.r-project.org/).

## Results

### Search Results and Study Characteristics

**Figure 1:**
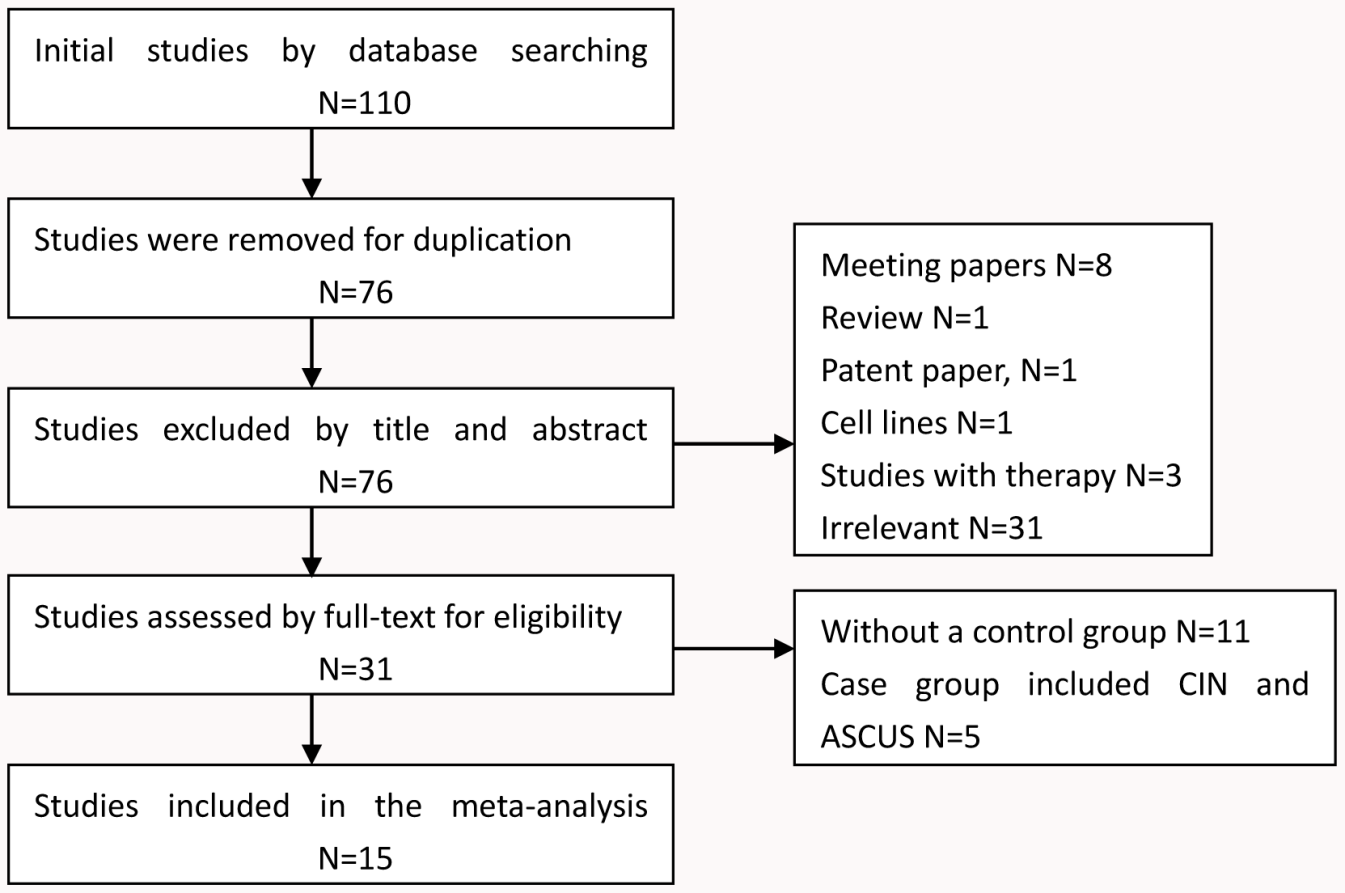
Flow chart of study selection for the meta-analysis.

A total of 15 studies that incorporated 1489 patients were included in this meta-analysis (Fig 1). In all, 110 studies were initially found after a search of the above databases, but 34 studies were excluded because of duplication. By screening the titles and abstracts of the remaining76 studies, a further 45 studies were excluded (8 meeting papers, 1 review, 1 patent paper, 1 cell lines, 3 studies with therapy, and 31 irrelevant articles). Eleven studies without a control group and 5 studies that included precancerous tissues such as ASCUS, LSIL, and HSIL in the case group, which meant that the raw data of the cancer patients could not be isolated, were excluded during the process of full-text review. Finally, 15 studies were included in this meta-analysis [3], [11]–[24]. The number of cases ranged from 22 to 350 among the studies with participants from, Asia (8 studies), North Africa (1 study), Europe and North America (7 studies). For the 15 studies, 5 studies used real-time quantitative polymerase chain reaction (QMSP) and the other 10 studies used methylation-specific polymerase chain reaction (MSP) to detect *DAPK1* methylation in the case group and in the control group (Table 1).

**Table 1:** Information of the studies included in the meta-analysis.

						Tumor	Control	
Author	Year	Country	Ethnicity	Method	Age (y)	M+/N	M+/N	Source of Controls
Sun et al. [21]	2012	China	Asian	MSP	39.3(18–65)	11/14	157/336	NT
Niyazi et al. [19]	2012	China	Asian	MSP	41.4(27–62)	19/30	1/30	BCT
Missaoui et al. [18]	2011	Tunisia	African	MSP	NA	10/14	0/8	BCT
Kim et al. [16]	2010	Korea	Asian	MSP	NA	50/69	11/41	BCT
Yang et al. [22]	2010	NE	Caucasian	QMSP	47(38–57)	18/60	5/20	BCT
Iliopoulos et al. [13]	2009	Greece	Caucasian	QMSP	41(18–62)	41/61	0/15	NT
Flatley et al. [3]	2009	UK	Caucasian	MSP	34.3(20–84)	17/42	0/40	NT
Zhao et al. [23]	2008	China	Asian	MSP	42(25–71)	34/52	0/20	BCT
Leung et al. [17]	2008	China	Asian	MSP	52.5(23–85)	60/107	0/27	AT
Feng et al. [12]	2007	USA	Caucasian	QMSP	46.8	31/63	1/16	NT
Shivapurkar et al. [20]	2007	USA	Caucasian	QMSP	NA	24/45	0/12	BCT
Jeong et al. [14]	2006	Korea	Asian	MSP	48.9(24–79)	35/78	1/24	BCT
Kang et al. [15]	2005	Korea	Asian	MSP	NA	60/82	0/17	BCT
Reesink-Peters et al. [24]	2004	NE	Caucasian	QMSP	NA	35/48	2/41	NT
Dong et al. [11]	2001	Korea	Asian	MSP	NA	27/53	0/24	BCT

Note: NE: Netherlands; UK: United Kingdom; MSP: methylation-specific polymerase chain reaction; QMSP: real-time quantitative methylation-specific polymerase chain reaction; NA: not applicable; M+: the number of methylations; N: number of total; NT: normal cervical tissues from healthy people; BCT: normal cervical tissues from patients who had benign gynecological diseases such as uterine myoma, adenomyoma, and uterine prolapse; AT: normal cervical tissues adjacent to the tumor.

### Quality assessment

The result of NOS demonstrated that the lowest score was 6 and highest score was 9 with a median score of 7.2. Most studies used healthy volunteers from the hospital as controls except those of Feng et al. [12] and Sun et al. [21]. The study by Leung et al. [17] was the only one where the control tissues were derived from adjacent normal tissues (Table 2).

**Table 2:** Quality assessment of included studies in the meta-analysis.

	Newcastle-Ottawa Scale									
Author	1	2	3	4	5	6	7	8	9	Total
Sun et al. [21]	Yes	Yes	Yes	Yes	Yes	Yes	Yes	Yes	Yes	9
Niyazi et al. [19]	Yes	Yes	NO	Yes	Yes	NO	Yes	Yes	Yes	7
Missaoui et al. [18]	Yes	Yes	NO	Yes	Yes	NO	Yes	Yes	Yes	7
Yang et al. [22]	Yes	Yes	NO	Yes	Yes	NO	Yes	Yes	Yes	7
kim et al. [16]	Yes	Yes	NO	Yes	Yes	Yes	Yes	Yes	Yes	8
Iliopoulos et al. [13]	Yes	Yes	NO	Yes	Yes	NO	Yes	Yes	Yes	7
Flatley et al. [3]	Yes	Yes	NO	Yes	Yes	NO	Yes	Yes	Yes	7
Zhao et al. [23]	Yes	Yes	NO	Yes	Yes	NO	Yes	Yes	Yes	7
Leung et al. [17]	Yes	Yes	NO	NO	Yes	NO	Yes	Yes	Yes	6
Feng et al. [12]	Yes	Yes	Yes	Yes	Yes	NO	Yes	Yes	Yes	8
Shivapurkar et al. [20]	Yes	Yes	NO	Yes	Yes	NO	Yes	Yes	Yes	7
Jeong et al. [14]	Yes	Yes	NO	Yes	Yes	NO	Yes	Yes	Yes	7
Kang et al. [15]	Yes	Yes	NO	Yes	Yes	NO	Yes	Yes	Yes	7
Reesink-Peters et al. [24]	Yes	Yes	NO	Yes	Yes	NO	Yes	Yes	Yes	7
Dong et al. [11]	Yes	Yes	NO	Yes	Yes	NO	Yes	Yes	Yes	7

1, indicates case definition and appropriate diagnosis; 2, consecutive patients or cases have a good representation; 3, community controls; 4, controls with no history of study disease; 5, according to the most important factor (patients in the control group were not diagnosed with cervical cancer or any other cancers) to select and analyze the controls; 6, according to the second most important factor (the age of the control group should not have significant heterogeneity compared with the case group) to select and analyze the controls; 7, ascertainment of exposure by blinded interview or record; 8, same method of ascertainment used for cases and controls; and 9, non-response rate the same for cases and controls.

### Meta-regression and subgroup analyses

**Figure 2:**
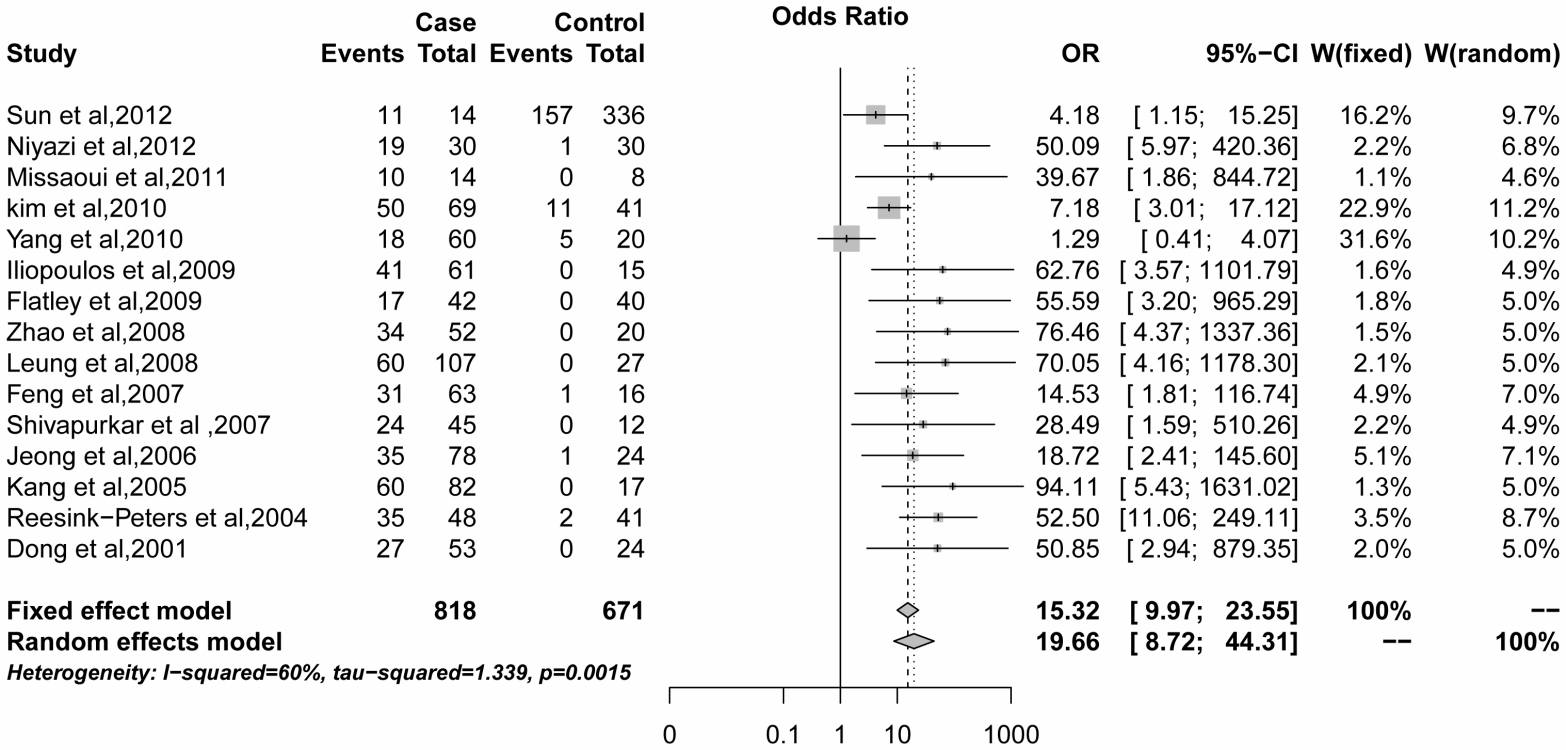
Pooled OR value for 15 selected studies.

The *x^2^*-based Cochran Q statistic test and *I* ^2^ statistics found significant heterogeneity among the 15 studies (*I^2^* = 60.0%, *P* = 0.002). A strong association was observed between *DAPK1* promoter methylation and cervical cancer with a pooled OR of  = 19.66 (95%CI = 8.72–44.31) based on the random effects model (Fig. 2). For this result, we tried to find the possible source of the heterogeneity. Based on previous studies and our present knowledge, we first used a multiple regression model with five variables based on publication year, ethnicity, method, source of controls, and case sample size. Case groups whose sample size was less than 60, were classified as group A, while the other groups were classified as group B. Through the regression model, we did not find a significant heterogeneity for the five variables listed above (Table 3). We then conducted a subgroup analysis to further assess the source of the heterogeneity.

**Table 3:** Meta-regression analysis on 15 selected studies (Table 1).

Sources of heterogeneity	Coefficient	95%CI		*P*
		Lower	Upper	
Publication year	1.2831	−0.3213	2.8875	0.1170
Ethnicity	1.2830	−2.5690	5.1351	0.5139
Method	−1.9797	−5.9054	1.9461	0.3230
Source of controls	0.3772	−1.3829	2.1373	0.6745
Case sample size	−0.7374	−2.1433	0.7964	0.3460

We performed a subgroup analysis according to ethnicity, method, and the case sample size. The OR was 18.22 in Caucasians (95%CI = 3.35–99.03; random effects model) and 17.88 (95%CI = 10.29–31.07; fixed effects model) in Non-Caucasians, the *I^2^* value were obtained separately and were determined to be 75.9% and 42.6% compared with the whole study group (*I^2^* = 60%). With this method, the ORs of the studies that used MSP was 19.10 (95%CI = 11.11–32.84; fixed effects model) and 15.30 (95%CI = 2.34–99.66; random effects model). Similarly, the OR in group A was 25.80 (95%CI = 12.56–53.02; fixed effects model) while the OR in group B was 13.55 (95%CI = 3.93–46.73; random effects model) (Table 4).

**Table 4:** Subgroup meta-analysis of the association between *DAPK1* promoter methylation and cervical cancer.

Group	Tumor		Control		M-H pooled OR	D+L pooled OR	Heterogeneity		
	M+	N	M+	N	OR(95%CI)	OR(96%CI)	*I^2^*(%)	*P*	τ2
Total	472	818	178	671	15.32(9.97–23.55)	19.66(8.72–44.31)	60.0	0.002	1.34
Ethnicity									
Non-Caucasians	306	499	170	527	17.88(10.29–31.07)	19.82(8.12–48.35)	42.6	0.083	0.70
Caucasians	166	319	8	144	12.27(6.25–24.10)	18.22(3.35–99.03)	75.9	<0.001	3.18
Method									
MSP	323	541	170	567	19.10(11.11–32.84)	21.07(8.98–49.43)	40.3	0.089	0.67
QMSP	149	277	8	104	10.46(5.21–21.01)	15.30(2.34–99.66)	78.8	<0.001	3.41
Sample size									
A	177	298	160	513	25.80(12.56–53.02)	27.25(10.67–69.58)	29.3	0.195	0.51
B	295	520	18	158	10.70(6.22–18.42)	13.55(3.93–46.73)	69.3	0.003	1.71

Notes: Non-Caucasians included Asians and Africans; A: The case sample size was less than 60; B: The case sample size was larger or equal to 60; M-H: the fixed effects mode; D+L: the random effects model.

### Sensitivity analysis and subgroup analyses

**Figure 3:**
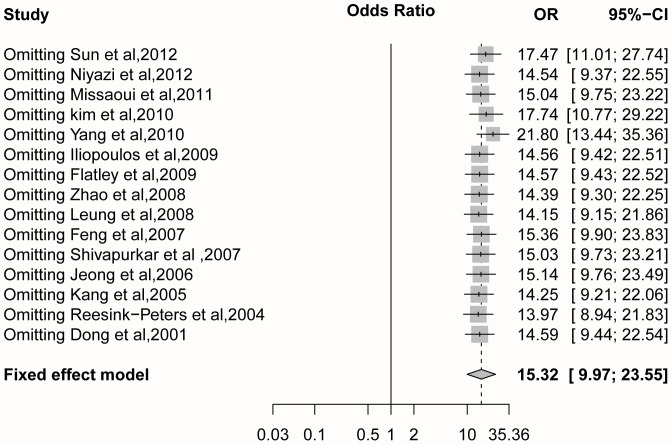
Sensitivity analysis of 15 studies with the fixed effects model.

**Figure 4:**
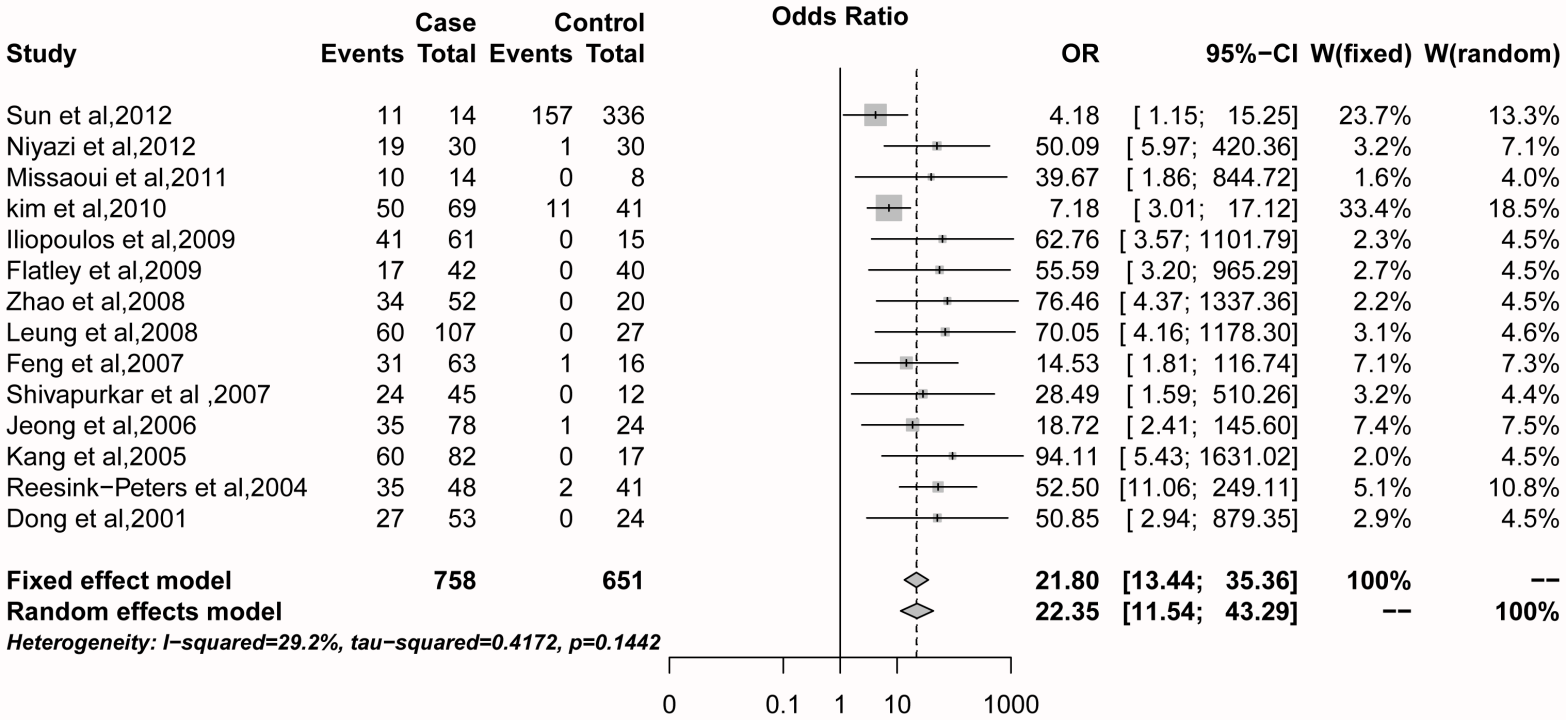
Pooled OR value of 14 studies omitting one heterogeneous study (Yang et al., 2010).

The result of the sensitivity analysis showed that the OR value ranged from 13.97 (95%CI = 8.94–21.83) to 21.80 (95%CI = 13.44–35.36) with a pooled OR of  = 15.32 (95%CI = 9.97–23.66) with the fixed effects model (Fig. 3). After the omission of the heterogeneous study (Yang et al., 2010), the pooled OR changed dramatically compared to when other studies were removed. Additionally, the initial heterogeneity (*I^2^* = 60.0%, *P* = 0.002) decreased to *I^2^* = 29.2% (*P* = 0.144) with a pooled OR of  = 21.80 (95%CI = 13.44–35.36; fixed effects model) when the heterogeneous study was removed (Yang et al., 2010) (Fig. 4). When, we made a further analysis based on ethnicity, method, and the case sample size, the results showed that the heterogeneity in Caucasians, QMSP method and larger sample size disappeared when the data from the heterogeneous study was removed (Yang et al., 2010) (Table 5).

**Table 5:** Subgroup meta-analysis of the association between *DAPK1* promoter methylation and cervical cancer omitting one heterogeneous study (Yang et al.).

Group	Tumor		Control		M-H pooled OR	D+L pooled OR	Heterogeneity		
	M+	N	M+	N	OR(95%CI)	OR(96%CI)	*I^2^*(%)	*P*	τ2
Total	454	758	173	651	21.80(13.44–35.36)	22.35(11.54–43.29)	29.2	0.144	0.42
Ethnicity									
Non-Caucasians	306	499	170	527	17.88(10.29–31.07)	19.82(8.12–48.35)	42.6	0.083	0.70
Caucasians	148	259	3	124	37.07(13.37–102.76)	37.44(13.82–101.40)	0.0	0.877	0.00
Method									
MSP	323	541	170	567	19.10(11.11–32.84)	21.07(8.98–49.43)	40.3	0.089	0.67
QMSP	131	217	3	104	34.29(11.53–101.97)	35.44(12.24–102.63)	0.0	0.771	0.00
Sample size									
A	177	298	160	513	25.80(12.56–53.02)	27.25(10.67–69.58)	29.3	0.195	0.51
B	277	460	13	138	18.57(9.63–35.81)	18.96(6.98–51.53)	32.7	0.191	0.49

Notes: Non-Caucasians included Asians and Africans; A: The case sample size was less than 60; B: The case sample size was larger or equal to 60; M-H: the fixed effects mode; D+L: the random effects model.

### Publication bias

**Figure 5:**
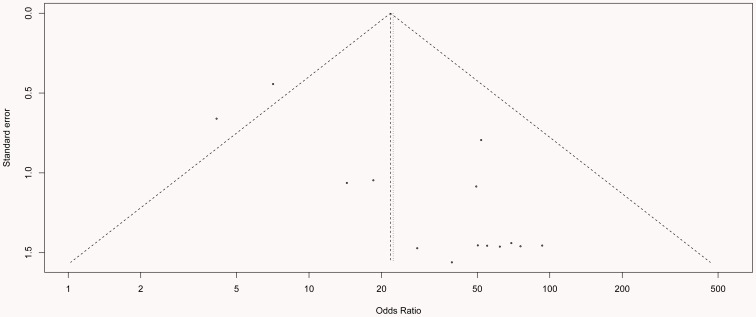
Egger's funnel plot for assessment of publication bias for the remaining 14 studies in the meta-analysis (each study is represented by a point).

Funnel plots and Egger's test were performed to assess the publication bias of the literature. The shape of the funnel plot in Figure 5 shows a possible asymmetry, but Egger's test resulted in *P* = 0.551, which indicates that publication bias was very low; no significant bias was found among the included studies. The fail-safe number (Z = 61.12, Nfs_0.05_ = 1374.98, Nfs_0.01_ = 674.13) also indicated that the degree of publication bias was very small.

## Discussion

Death-associated protein kinase1 (*DAPK1*) could mediate cell death via IFN-gamma and could lead to tumor pathogenesis and metastasis when inactivated [25]. Recently, many studies have shown that DNA methylation alterations are involved in cancer initiation and progression, and could be used to predict the diagnosis and prognosis of human diseases and malignancies [26], [27]. The loss of *DAPK1* expression, mainly by hypermethylation of its promoter region, enhances the metastatic potential of cancer cells and has been proven to occur in a variety of cancers, including cancer of the uterine cervix [14], [24]. Although HPV infection is one of the most important risk factors, the majority of patients with HPV infection do not develop cervical cancer. HPV infection alone is insufficient for malignant transformation of cervical cells, which suggest potential roles of other genetic and epigenetic events in cervical carcinogenesis [11].

the result of the pooled OR was 19.66 (95%CI = 8.72–44.31) with the random effects model (Fig 1), which showed that *DAPK1* promoter methylation is associated with cervical cancer and therefore, that it might play an important role in the pathogenesis of cervical cancer. This result was consistent with the findings of previous studies [11], [20]. However, significant heterogeneity was observed in those 15 studies, and the reason for heterogeneity could not be explained at the beginning. To explore the possible source of the heterogeneity, we implemented a meta-regression and subgroup analysis. The results showed some heterogeneity in Caucasians, the QMSP method and in a larger sample size through the subgroup analysis (Table 4); then, we conducted a sensitivity analysis to find the source of the heterogeneity. The study by Yang et al. (2010) seems to be the heterogeneous study that affected the meta-analysis, as *I^2^* = 60% (*P* = 0.002) was reduced to *I^2^* = 29.2% (*P* = 0.144) when this study was omitted (Fig 4). In addition, further statistical analysis confirmed the heterogeneity of the study of Yang et al. (2010), and no significant heterogeneity of the remaining 14 studies (Table 5).

When the heterogeneous study was omitted, the pooled OR value was increased from 15.32 to 21.80 (the fixed effects model), which suggested a stronger association between *DAPK1* promoter methylation and cervical cancer. The heterogeneity presented in QMSP was decreased from *I^2^* = 78.8% to *I^2^* = 0.0%, which indicated that the method of QMSP is better than that of MSP. This conclusion was consistent with the study by Eads et al. [28]. The heterogeneity in Caucasians also decreased from *I^2^* = 75.9% to *I^2^* = 0.0%, which might have been caused by two major reasons. First, the detection of *DAPK1* promoter methylation in Caucasians by the QMSP method excluded the study by Flatley et al.. The other reason may be that the heterogeneity in Caucasians is relatively small. Publication bias was evaluated through Funnel plots and Egger's test, and the Egger's test showed *P* = 0.551, which indicated that there was no significant publication bias. The fail-safe number further confirmed that the trend for publication bias was very small.

This meta-analysis had some limitations. The first limitation was that some studies did not provide detailed information regarding the age of individuals in the case groups and control groups. The second limitation in this meta-analysis was that some studies did not reveal the stage of the cervical cancers or the subtype, which might also be sources of the heterogeneity. Considering the small number of articles that described the stage and type of cervical cancer, the power was too small to make a subgroup for them. Other confounding variables such as method, ethnicity, sample size, and the source of control may also exist. Publication bias was the third limitation. Some unpublished and negative studies may contribute to some bias though no significant publication bias was detected according to Egger's test.

In conclusion, a strong association was observed between *DAPK1* promoter methylation and cervical cancer, and therefore, *DAPK1* promoter methylation may be valuable as a biomarker. Considering that the quality and quantity of the reviewed articles were limited, larger and well-designed studies should be employed in the future for further confirmation of the association between *DAPK1* promoter methylation and cervical cancer.

## Supporting Information

Checklist S1 PRISMA Checklist.
(DOC)
